# A Novel Approach to Global Positioning System Accuracy Assessment, Verified on LiDAR Alignment of One Million Kilometers at a Continent Scale, as a Foundation for Autonomous DRIVING Safety Analysis

**DOI:** 10.3390/s21175691

**Published:** 2021-08-24

**Authors:** Janusz Bedkowski, Hubert Nowak, Blazej Kubiak, Witold Studzinski, Maciej Janeczek, Szymon Karas, Adam Kopaczewski, Przemyslaw Makosiej, Jaroslaw Koszuk, Michal Pec, Krzysztof Miksa

**Affiliations:** TomTom International BV, 1011 AC Amsterdam, The Netherlands; Hubert.Nowak@tomtom.com (H.N.); Blazej.Kubiak@tomtom.com (B.K.); Witold.Studzinski@tomtom.com (W.S.); Maciej.Janeczek@tomtom.com (M.J.); Szymon.Karas@tomtom.com (S.K.); Adam.Kopaczewski@tomtom.com (A.K.); Przemyslaw.Makosiej@tomtom.com (P.M.); Jaroslaw.Koszuk@tomtom.com (J.K.); Michal.Pec@tomtom.com (M.P.); Krzysztof.Miksa@tomtom.com (K.M.)

**Keywords:** global positioning, SLAM, GNSS + INS, road survey, mobile mapping, autonomous driving safety

## Abstract

This paper concerns a new methodology for accuracy assessment of GPS (Global Positioning System) verified experimentally with LiDAR (Light Detection and Ranging) data alignment at continent scale for autonomous driving safety analysis. Accuracy of an autonomous driving vehicle positioning within a lane on the road is one of the key safety considerations and the main focus of this paper. The accuracy of GPS positioning is checked by comparing it with mobile mapping tracks in the recorded high-definition source. The aim of the comparison is to see if the GPS positioning remains accurate up to the dimensions of the lane where the vehicle is driving. The goal is to align all the available LiDAR car trajectories to confirm the of accuracy of GNSS + INS (Global Navigation Satellite System + Inertial Navigation System). For this reason, the use of LiDAR metric measurements for data alignment implemented using SLAM (Simultaneous Localization and Mapping) was investigated, assuring no systematic drift by applying GNSS+INS constraints. The methodology was verified experimentally using arbitrarily chosen measurement instruments (NovAtel GNSS + INS, Velodyne HDL32 LiDAR) mounted onto mobile mapping systems. The accuracy was assessed and confirmed by the alignment of 32,785 trajectories with a total length of 1,159,956.9 km and a total of 186.4 × 10${}^{9}$ optimized parameters (six degrees of freedom of poses) that cover the United States region in the 2016–2019 period. The alignment improves the trajectories; thus the final map is consistent. The proposed methodology extends the existing methods of global positioning system accuracy assessment, focusing on realistic environmental and driving conditions. The impact of global positioning system accuracy on autonomous car safety is discussed. It is shown that 99% of the assessed data satisfy the safety requirements (driving within lanes of 3.6 m) for Mid-Size (width 1.85 m, length 4.87 m) vehicles and 95% for Six-Wheel Pickup (width 2.03–2.43 m, length 5.32–6.76 m). The conclusion is that this methodology has great potential for global positioning accuracy assessment at the global scale for autonomous driving applications. LiDAR data alignment is introduced as a novel approach to GNSS + INS accuracy confirmation. Further research is needed to solve the identified challenges.

## 1. Introduction

**Figure 1:**
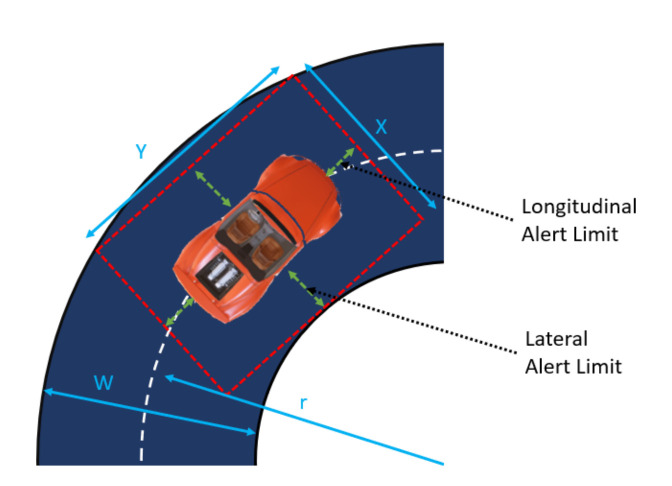
Bounding box geometry during a turn maneuver. This shows the allowable maximum position error of the vehicle to ensure it is within the lane known as the alert limits [1].

*Problem statement*: The goal of the presented research is to measure the impact of the global positioning system on the autonomous driving safety. The challenge of the research is to assess the positioning accuracy of cars moving onto limited-access highways in the USA. Localization accuracy requirements for US freeway operation are discussed in [1]. Due to the nature of the measurement, it is difficult to perform repeatable data collections since cars never follow the same trajectories. The actual coverage limits the possibility of repetitive measurements and introduces an important challenge being the lack of the ground truth data. Thus, accuracy assessment is the main focus of the paper, and it requires a new approach that is formulated as a novel measurement methodology. Safety of autonomous driving is addressed as an alert limit for the defined geometry of the problem, where the aim is to maintain knowledge that the vehicle (its bounding box) is within its lane. Horizontally, this is expressed as lateral (side-to-side) and longitudinal (forward–backward) components. Vertically, the vehicle must know what road level it is on (location among multi-level roads). The relationship between the road width and curvature and the bounding box around the vehicle is shown in Figure 1.

The relationship between the lateral *x* and longitudinal *y* bounds and road geometry *w* is defined in Equation (1).

(1) $$x=\sqrt{{\left(r+\frac{w}{2}\right)}^{2}-{\left(\frac{y}{2}\right)}^{2}}+\frac{w}{2}-r$$

The authors of [1] define alert limits related to the vehicle length $l_{v}$ and width $w_{v}$ as (2):

(2) $$\begin{array}{c} Lateral\;Alert\;Limit=\frac{x-w_{v}}{2}\\ Longitudinal\;Alert\;Limit=\frac{y-l_{v}}{2} \end{array}$$

For the impact of GNSS positioning on safety, the following aspects are considered: vehicle type, a mean distance between lanes of 3.6 m (limited access highways in the United States of America), Lateral Alert Limit and Longitudinal Alert Limit. Reference alert limits of relative positioning for different types of vehicle versus map are shown in Table 1 [1].

*Problem formulation*: The problem is to confirm the global positioning system accuracy assessed in our case by state-of-the-art NovAtel algorithm and relate it with the autonomous driving safety. We investigated how to use LiDAR metric measurements to align all available trajectories. Data were collected using a mobile mapping road survey performed at a continent scale. Based on this data collection, many challenges were determined and addressed in the paper. Some of the challenges are the following: large area coverage, the impact of environmental conditions, and dynamic changes of road geometry like roadworks. The most important requirement for calculating alignment is to ensure no systematic drift of aligned trajectories. Thus, the alignment method should work by means of Least Squares using assessed trajectories as constraints. Another problem is to maintain the shape of aligned trajectories; thus, the motion model must constrain all relative consecutive poses. Bearing in mind the requirements for aligned trajectories, the result is optimal; therefore, the confirmation of the assessment accuracy of the trajectories can be applied. It can only be achieved by means of massive data processing performed to obtain quantitatively correct results.

*Problem assessment*: A new methodology is proposed for global positioning system accuracy assessment to analyze the impact on autonomous driving safety. It is composed of six elements:

- Mobile mapping system minimal setup;
- Global positioning data processing;
- LiDAR data processing;
- Alignment algorithm;
- Accuracy assessment confirmation;
- Autonomous driving safety analysis.

**Figure 2:**
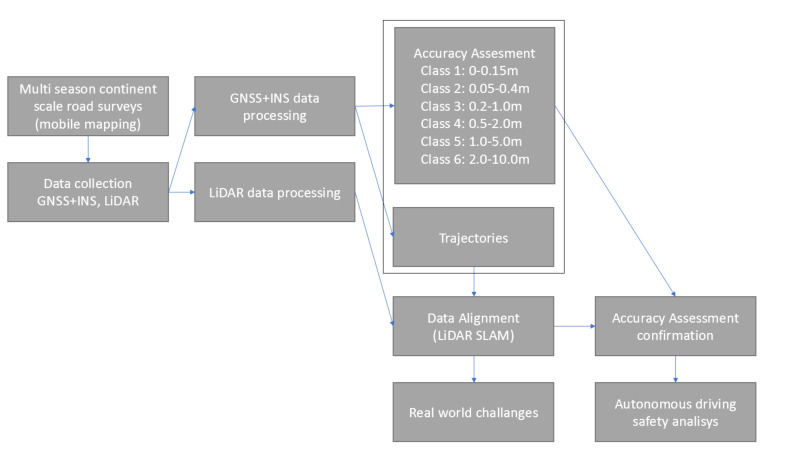
The scheme of GNSS + INS accuracy assessment for autonomous driving safety analysis.

The scheme of the experimental verification is shown in Figure 2.

The GNSS + INS accuracy of fast-moving vehicles was measured at a large scale, covering as much of the limited-access highways in the USA as possible, as realistic dynamic conditions are considered a core requirement. GNSS + INS NovAtel was chosen as a reference global positioning measurement instrument mounted on mobile mapping systems equipped with a single Velodyne HDL32 3D LiDAR. GNSS receivers are integrated with mobile mapping systems, and the measurements are post-processed using a combination of NovAtel PPP (Precise Point Positioning) and PPK (Post-Processed Kinematic) algorithms, thus obtaining the most accurate positioning from the point of view of an applied measurement instrument. To reach satisfactory results, it was decided to use mobile mapping data covering most of the limited access highways in the USA. *The aim of experimental verification of the proposed methodology is to use GNSS + INS trajectories as objects of accuracy assessment, align them using LiDAR data, confirm the accuracy and perform autonomous driving safety analysis.* This is possible only if it is ensured that the alignment does not introduce any systematic drift. For this reason, the use of the state-of-the-art LiDAR SLAM algorithm was investigated. The algorithm is based on the Weighted Nonlinear Least-Square Method capable of aligning these trajectories based on LiDAR observations, motion model and GNSS + INS constraints. Based on this investigation, some deviations in the accuracy of GNSS + INS are demonstrated. It is a very important research topic since the era of autonomous driving is approaching. The challenges related to the proposed methodology are as follows. The first challenge is that there is no ground truth for such a scope of data. Moreover, accurate tracking of the fleet of fast-moving mobile mapping systems is impossible considering the continent-scale coverage. The second challenge is enuring no systematic drift in the aligning procedure. The third challenge is related to many factors affecting the alignment algorithm relying on LiDAR measurements. The fourth challenge is related to dynamic conditions of the data collection and many environmental changes (e.g., roadworks, weather conditions) that could affect LiDAR-based alignment.

The main requirement is to collect large-scale, mobile mapping data (LiDAR, GNSS + INS) covering as large area as possible to ensure LiDAR data overlapping. It is advised to use multiple mobile mapping systems with the same setup of the measurement instruments. Thus, the results of the experiments are not affected by the bias of using only one measurement instrument. This paper addresses an approach for the continent-scale SLAM experiment, which is a contribution to the Mobile Robotics domain where the large scale is an interesting research topic. This is an important research topic from the perspective of recent developments in the localization of autonomous cars [2,3]. It is evident that autonomous cars can collect data and contribute to global map updates; thus, it is a large-scale problem that inspires many researchers.

The term SLAM [4] corresponds to a “chicken and egg dilemma”. It is therefore necessary to have a proper map representation that is compatible with observations derived from sensors to localize the vehicle within the map, and accurate localization is needed to build the map. The core concept is the pose that represents position and orientation at a given time. A set of consecutive poses makes up a trajectory. Attaching measurements to the trajectory as relative poses gives an opportunity to reconstruct a map of raw measurements, e.g., the point cloud in the case of using LiDAR technology. The calibration parameters also must be considered to ensure proper transformation from the trajectory pose to the sensor origin.

This paper concerns the concepts and methods known from Mobile Robotics and Geodesy domains. These domains introduce a methodology for map building based on computing the absolute pose of measurement instruments assuming raw information typically transformed into feature space [5]. It addresses how to fill the gap between these domains, which is also discussed in [6]. Therefore, the problem of fusing GNSS + INS and LiDAR observations to align all trajectories ensuring no systematic drift is the main research topic discussed in this paper. The result of this research is a new methodology of GNSS + INS accuracy assessment. The paper is organized as follows: Section 2 discusses the state of the art related to mobile mapping approaches and available data sets. Section 3 concerns an experimental verification of the proposed methodology and defines the minimal setup of mobile mapping systems, GNSS + INS data processing, LiDAR data processing, SLAM algorithm ensuring no systematic drift of aligned trajectories and impact on autonomous driving safety. Section 4 addresses real-world challenges affecting data alignment, providing important feedback for the research community. In Section 5, experimental validation details are provided, and the results are discussed in Section 5.2. The impact of GNSS + INS positioning on autonomous driving safety is elaborated in Section 6. Final conclusions are given in Section 7.

## 2. State of the Art

Trajectory, sensor readings and map are terms commonly used in Mobile Robotics in the context of SLAM. The trajectory can be expressed as consecutive 6-DOF poses [7]. Collecting consistent 3D laser data using a moving mobile mapping system is often difficult because the precision of collected data is related to motion estimation. For this reason, the trajectory of the sensor during the scan must be taken into account while constructing 3D point clouds. To address this issue, many researchers use the stop-scan fashion—they stop a moving platform and take stationary scans [8,9]. On the contrary, in recent research advances, continuous-time mapping is favored [10,11]. Continuous-time mapping relates to the new term of the time calibration method [12], and it introduces a continuous-time, simultaneous localization and a mapping approach for mobile robotics. In comparison, mobile mapping systems used in geodesy use synchronized sensor readings.

Mobile Mapping Systems are composed of proprioceptive, exteroceptive and interoceptive sensors. Proprioceptive sensors measure the internal state of the system in the environment such as position, velocity, acceleration and temperature. Exteroceptive sensors measure parameters external to the system such as pressure, forces and torques, vision, proximity and active ranging. Vision sensors include monocular, stereo/multiple cameras, equirectangular/spherical cameras and structured lighting (e.g., so-called RGBD cameras). There are active ranging systems laser line scanners, such as LiDAR, RADAR and SONAR. Interoceptive sensors measure electrical properties (voltage, current), temperature, battery charge state, stress/strain and sound. All above-mentioned sensors are connected to the dedicated electronics that synchronize all inputs with the GNSS receiver; thus all raw data can be transformed into global reference systems. There is a need to cope with GNSS-denied environments; thus, recent developments show the progress of mobile mapping technologies that also use SLAM algorithms. A mobile mapping device capable of building the map was introduced in [13]. Such devices use the advantage of a rotating LiDAR to perceive full 360-degree distance measurements. Further developments introduce equirectangular cameras that can augment metric information with spherical images. Many mobile mapping applications incorporate equirectangular camera FLiR Ladybug5/5+ to perceive 360-degree spherical images [14]. High-end mobile mapping systems [15,16] use more precise measurement instruments, which involve a higher cost.

### 2.1. Large-Scale Data Sets

In recent research since mobile mapping systems became more affordable, many open-source large data sets have appeared. The GNSS-specific data set [17] contains GNSS data from two sensors recorded during real-world urban driving scenarios. A mass-market receiver is used, and the ground truth is derived from a highly accurate reference receiver. The complex urban data set [18] provides LiDAR data and stereo images with various position sensors targeting a highly complex urban environment. It captures features in urban environments (e.g., metropolitan areas, complex buildings and residential areas). The data of 2D and 3D of LiDAR is provided. Raw sensor data for vehicle navigation and development tools are given in a ROS file format.

Authors of the Multi Vehicle Stereo Event Dataset [19] provide a collection of data helpful in the development of the 3D perception algorithms for event-based cameras. An interesting data set [20] includes data from the AtlantikSolar UAV (Unmanned Aerial Vehicle), which is a small-sized, hand-launchable, solar-powered device optimized for large-scale aerial mapping and inspection applications. Authors of [21] provide the Oxford RobotCar data set that contains over 100 repetitions of a consistent route through Oxford, United Kingdom, captured throughout over a year. Additionally, the authors provide RTK Ground Truth [22]. The authors of [23] provided the Málaga Stereo and Laser Urban Data Set, which was gathered in urban scenarios with a car equipped with a stereo camera (Bumblebee2) and five LiDARs. The KITTI-360 data set [24], which is well-known in Mobile Robotics and Machine Vision domains, include data from an autonomous driving platform called Annieway.

### 2.2. Long-Term Data Sets

Long-term data sets include multi-season data. The purpose is to address the impact of multi-season, varying weather and other disturbances into localization algorithms. The authors of [25] provided the KAIST multi-spectral data set that covers regions from urban to residential for autonomous systems. They claim that this data set provides different perspectives of the world captured in coarse time slots (day and night) in addition to fine time slots (sunrise, morning, afternoon, sunset, night and dawn). The interesting Visual-Inertial Canoe data set [26] includes data from a canoe along the Sangamon River in Illinois. The authors state that the canoe was equipped with a stereo camera, an IMU and a GPS device, which provide visual data suitable for stereo or monocular applications, inertial measurements and position data for the ground truth. University of Michigan North Campus Long-Term (NCLT) Vision and LiDAR Dataset [27] consists of omnidirectional (equirectangular) imagery, 3D LiDAR, planar LiDAR, GPS and proprioceptive sensors for odometry collected using a Segway robot. The authors conducted this research to allow researchers to focus on long-term autonomous operation in changing environments. Lyft’s [28] Level 5 Perception Dataset 2020 is relevant as both a large-scale and long-term data set. It is maintained by autonomous vehicles that collect raw sensor data on other cars, pedestrians, traffic lights and more. This data set features raw LiDAR and camera inputs collected by the autonomous fleet within a bounded geographic area.

### 2.3. Large-Scale Surveying and Mapping

Large-scale surveying and mapping relate to the shape of the Earth and spatial relations between objects near its surface. It is evident that global and local coordinate systems are useful for calculations. To describe the position in the global reference system (global geocentric terrestrial system), the coordinates are defined with respect to center of the Earth. Spatial relations between objects can be described using a local reference system. 3D Cartesian geocentric coordinates are not very convenient for describing positions on the surface of the Earth. It is more convenient to project the curved surface of the Earth onto a flat plane, which is related to map projections. Usually, the local coordinate system has the y-axis pointing in the North direction, the z-axis in the up direction, and the x-axis completing the pair and therefore pointing in the East direction. This type of system is referred to as a topocentric coordinate system. For the coordinates, it is common to use the capital letters ENU instead of x, y, z [29], and these are called Local Tangent Plane Coordinates. The alternative way of expressing the z-coordinate as a positive number (convenient for aeroplanes) is NED. All observation equations described in this paper are expressed in the right-handed local 3D Cartesian coordinate system; therefore, it is important to keep in mind the transformation function from the local to global coordinate system looking at the GPS data used for georeferencing [30].

Rigid transformation in **SE**(3) can be separated into two parts: translation and rigid rotation. There are plenty ways to express rotations [31,32] such as using Tait–Bryan and Euler angles, Quaternions [33], Rodriguez [34,35,36] and, e.g., Cayley formula [37]. Further information on how to construct transformation matrices can be found in [38,39,40]. The information on how to compute derivatives for rotations can be found in [41,42].

## 3. Experimental Verification of the Methodology

### 3.1. Mobile Mapping System Minimal Setup

**Figure 3:**
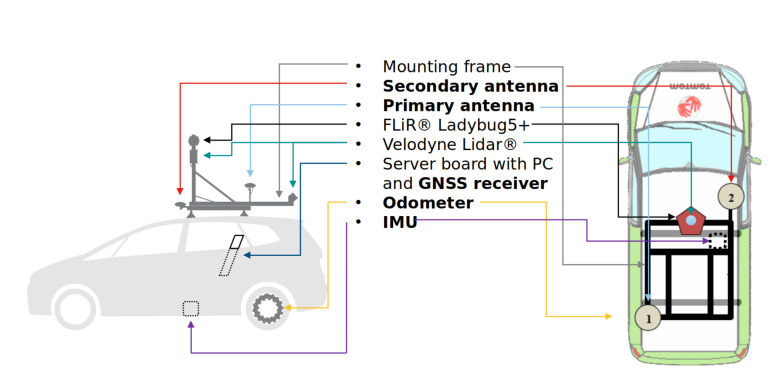
MoMa van—a TomTom B.V. Mobile Mapping proprietary technology—providing calibrated data.

The minimal setup of the mobile mapping system is at least one 3D LiDAR, GNSS + INS positioning system and odometry. To assess positioning systems other than GNSS + INS, an additional measurement instrument should be integrated with the mobile mapping data acquisition pipeline. All data should be synchronized. An example of such a mobile mapping system is the MoMa (Mobile Mapping) van—TomTom B.V. proprietary technology—is shown in Figure 3. It is composed of NovAtel ProPak6®/PwrPak7 GNSS receiver, NovAtel VEXXIS GNSS-850 GNSS antennas, ADIS 16488/KVH 1750 Inertial Measurement Unit, DIY odometer, Velodyne Lidar HDL-32E and FLiR Ladybug 5/5+ LD5P. All data are synchronized, and the relative poses of all sensors are obtained from in-house calibration procedure.

### 3.2. GNSS + INS Data Processing

**Figure 4:**
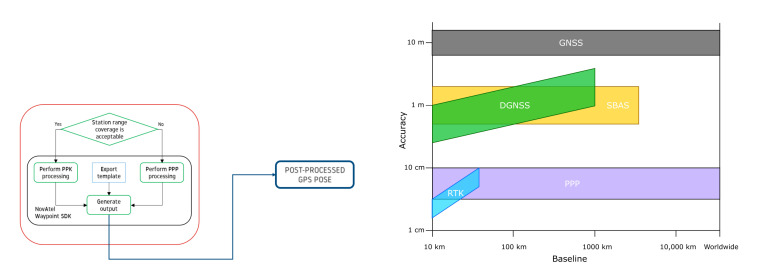
Diagram from “Precise Positioning with NovAtel CORRECT Including Performance Analysis” released in 2015 by NovAtel Inc.

GNSS + INS measurements are post-processed using a combination of NovaTel PPP (Precise Point Positioning) and PPK (Post-Processed Kinematic) algorithms shown in Figure 2 (left). All data are processed by NovaTel Waypoint Post-Processing Software SDK (Software Development Kit) 8.90 [43]. PPK and PPP methods incorporate information from GLONASS Satellite Constellation, Satellite Constellation, Geostationary Satellite (GEO) and Reference Stations [44]. The expected accuracy is shown in Figure 4 (right). Due to fact that PPK relates to RTK (Real-Time Kinematic), this method can reach much higher precision compared to PPP. NovAtel introduces six classes of accuracy, as shown in Figure 2. For the experiment purpose, all post-processed GNSS + INS data were transformed to ITRF2008 epoch 2019.0000.

### 3.3. Lidar Data Processing

**Figure 5:**
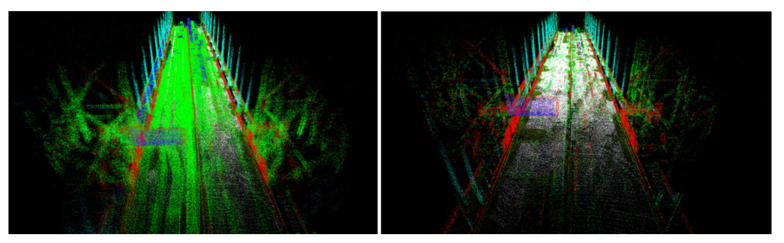
(**Left**)—point cloud affected by noise from traffic; (**Right**)—filtered and classified LiDAR data.

3D data derived from Velodyne HDL-32 utilize 32 LiDAR channels aligned from +10.67 to −30.67 degrees to provide an unmatched vertical field of view and a real-time 360-degree horizontal field of view. They generate a point cloud of up to 695,000 points per second with a range of up to 100 m and typical accuracy of ±2 cm. Reflectivity is used (values 0–255), and 3D coordinates of the measured points in Euclidean space are represented as (x, y, z). In this particular application, 3D data are downsampled for equal 3D points distribution and filtered for traffic noise reduction. The remaining point cloud is distinguished into basic primitives (point, cylindrical, plane) and assigned semantic labels related to reflectivity. Therefore, the result is a set of points with high reflectivity, points with low reflectivity, lines with high reflectivity, lines with low reflectivity, planes with high reflectivity, and planes with low reflectivity. This segmentation allows matching similar primitives as corresponding landmarks. To distinguish primitives of high and low reflectivity, and empirically estimated threshold is used; thus, based on empirical experiments, 3D points with reflectivity of more than 40 are considered as highly reflective and others as having low reflectivity. Traffic noise is a challenging aspect, since most of the road surveys were performed in realistic conditions; thus, RANSAC (Random Sample Consensus) [45] was applied for extracting surface planes. This method efficiently identifies surface planes even for a large volume of noisy traffic data (Figure 5-left) and the relevant implementation is available in PCL (Point Cloud Library) [46].

When data are downsampled and filtered, the grouping of points into basic primitives as lines, cylinders and planes is introduced, assuming a low–high reflective threshold (Figure 5: right). The result of this classification is the semantic label *l* assigned for each query point. In that sense, the impact of perceptual aliasing confusion [47] is addressed; thus the issue related to outlier observations (incorrectly matched landmarks) is addressed. In the literature, there are many techniques for automatic classification of point clouds such as semantic Classification of 3D Point Clouds with Multiscale Spherical Neighborhoods [48] that uses local features for classification. Another interesting technique—contour detection in unstructured 3D point clouds—was elaborated in [49]. In our application, an additional basic primitive as the direction of the line and the normal vector of the plane are calculated and used for constructing observation equations. For calculating the direction of the line and the normal vector of the plane, the following covariance matrix is used:

(3) $$C\left(N_{R}\right)=\frac{1}{N}\sum_{p\in N}^{}\left(p-\bar{p}\right){\left(p-\bar{p}\right)}^{T}$$

Its eigen-values $\lambda_{1}⩾\lambda_{2}⩾\lambda_{3}\in R$ and corresponding eigenvectors $e_{1},e_{2},e_{3}\in R^{3}$, and *N* is the number of points *p* found in certain radius *R* and $\bar{p}$ is the centroid of the neighborhood $N_{R}$ (all points inside the sphere of radius = *R*). The eigen-values and eigen-vectors are used for local shape description (linearity—Equation (4); planarity—Equation (5)) similar as in [7].

(4) $$Linearity=\left(\lambda_{1}-\lambda_{2}\right)/\lambda_{1}$$

(5) $$Planarity=\left(\lambda_{2}-\lambda_{3}\right)/\lambda_{1}$$

The implementation details are available in the form of point cloud processing tutorial available in [50].

### 3.4. Alignment Algorithm

The goal is to find an optimal solution for the desired poses of all GNSS + INS trajectories acquired with MoMa vans assuming information from LiDAR. The problem is formulated using the Weighted Nonlinear Least Square method, a special case of Generalized Least Squares, known, e.g., in photogrammetry [51] and LiDAR data matching [52]. The SLAM problem is nonlinear [5] due to rotations; therefore, a first-order Taylor expansion is used to construct the design matrix **A**. More information concerning observations and the Least Square method can be found in [53,54]. It is assumed that observational errors are uncorrelated; thus the weight matrix **P** is diagonal, and the problem becomes

(6) $$\left(A^{T}PA\right)\Delta x=A^{T}Pb$$

Larger values of elements in **P** determine the higher impact of the observation equation on the optimization process. A similar approach can be found in work on continuous 3D scan matching [11], where authors additionally incorporated a Cauchy function applied to the residuals **b** to cope with outliers. To solve a single iteration such as

(7) $$\Delta x={\left(A^{T}PA\right)}^{-1}\left(A^{T}Pb\right)$$

the sparse Cholesky factorization [55] is used. More implementation details concerning semantic data registration are available as Lesson 16 of the tutorial [50].

Rotation matrix representation such as Tait–Bryan angles [40] is used. Angles associated with the sequence (x, y, z) correspond to (ω, φ, κ) as (roll, pitch, yaw). They are commonly used in aerospace engineering and computer graphics. In the three-dimensional space, the following rotations via each axis are given:

(8) $$R_{x}\left(\omega \right)=(\begin{array}{ccc} 1 & 0 & 0\\ 0 & cos(\omega ) & -sin(\omega )\\ 0 & sin(\omega ) & cos(\omega ) \end{array}),R_{y}\left(\varphi \right)=(\begin{array}{ccc} cos(\varphi ) & 0 & sin(\varphi )\\ 0 & 1 & 0\\ -sin(\varphi ) & 0 & cos(\varphi ) \end{array}),R_{z}\left(\kappa \right)=(\begin{array}{ccc} cos(\kappa ) & -sin(\kappa ) & 0\\ sin(\kappa ) & cos(\kappa ) & 0\\ 0 & 0 & 1 \end{array})$$

Therefore, rotation matrix **R** is expressed as:

(9) $$R_{\omega \varphi \kappa }=(\begin{array}{ccc} \begin{array}{c} cos(\varphi )cos(\kappa ) \end{array} & -cos(\varphi )sin(\kappa ) & sin(\varphi )\\ cos(\omega )sin(\kappa )+sin(\omega )sin(\varphi )cos(\kappa ) & cos(\omega )cos(\kappa )-sin(\omega )sin(\varphi )sin(\kappa ) & -sin(\omega )cos(\varphi )\\ sin(\omega )sin(\kappa )-cos(\omega )sin(\varphi )cos(\kappa ) & sin(\omega )cos(\kappa )+cos(\omega )sin(\varphi )sin(\kappa ) & cos(\omega )cos(\varphi ) \end{array})$$

Finally, the optimization problem concerns finding updates $\Delta x_{ij}$ for all trajectory poses composed of six parameters including translation part (x, y, z) and rotation part (ω,φ,κ)

(10) $$\Delta x_{ij}=\left(\Delta x_{ij},\Delta y_{ij},\Delta z_{ij},\Delta \omega_{ij},\Delta \varphi_{ij},\Delta \kappa_{ij}\right)$$

where *i* corresponds to the $i^{th}$ trajectory and *j* corresponds to the $j^{th}$ pose.

In the proposed methodology, the required observation equations forming the SLAM alignment are defined as follows

- Semantic point-to-point (Section 3.4.1).
- Semantic point-to-projection (Section 3.4.2).
- Motion model and GNSS + INS as relative poses constraints (Section 3.4.3).

**Figure 6:**
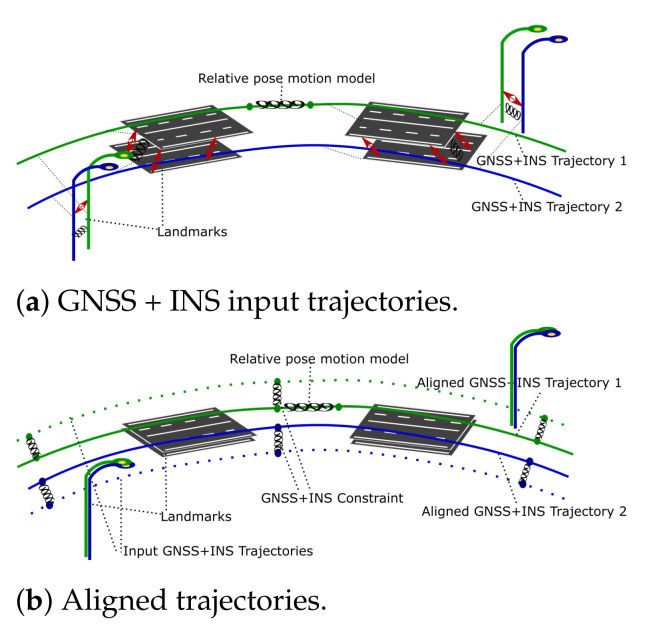
The idea of aligning trajectories assuring no systematic drift by incorporating GNSS + INS input data as the constraints. Springs visualize the constraints.

**Figure 7:**
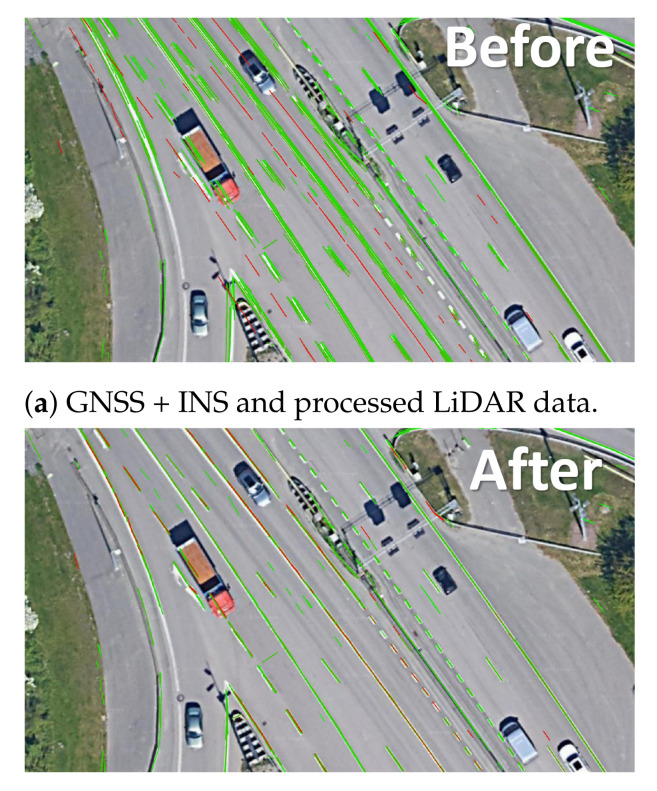
Result of the alignment, green—accurate data, red—inaccurate data.

Similar approaches can be found in [7,11,56,57,58,59] and the implementation of SLAM [60]. It is worth mentioning another family of observation equations that corresponds to local geometric features-called surfels in [11]. This particular application of SLAM has to ensure no systematic drift of aligned trajectories. For this reason, assessed GNSS + INS input trajectories are treated as constraints implemented using relative pose observation Equation (Section 3.4.3). This means that the desired relative pose $P^{t}$(x,y,z,ω,φ,κ) between the input GNSS + INS trajectory node and aligned one is $P^{t}$(0,0,0,0,0,0). Another important aspect of the proposed methodology requires no change in the shape of aligned trajectories; thus the motion model (as consecutive relative poses of GNSS + INS input trajectories) is used as a constraint and is also implemented as relative pose observation equation. In this case, the desired relative pose between consecutive nodes of aligned trajectories is calculated from GNSS + INS input trajectories and constrains the optimization process. This approach guarantees a similar shape of aligned trajectories to the input data, which is crucial for our application. In this sense, the optimization process will try to maintain the shape, positions and orientations of all input trajectories. All the LiDAR-based observation equations can affect the above-mentioned constraints to minimize the displacement of corresponding landmarks observed from different viewpoints. The idea is presented in Figure 6, and an example of data alignment is shown in Figure 7.

#### 3.4.1. Semantic Point-to-Point Observation Equation

The raw LiDAR measurement is represented as source point $P^{s}$($x^{s}$,$y^{s}$,$z^{s}$) in Euclidean space as point in a local reference frame. The matrix [**R**,T] is the transformation of source point $P^{s}$ into target point $P^{t}$($x^{t}$,$y^{t}$,$z^{t}$) in global reference frame; thus

(11) $$\Psi_{R,T}\left(x^{s},y^{s},z^{s}\right)=P^{t}=\left[R,T\right]P^{s}$$

The transformation [**R**,T] has a unique representation as a pose (x,y,z,ω,φ,κ), composed of position (x,y,z) and orientation (ω,φ,κ). Orientation corresponds to Tait–Bryan angles, respectively, ω:x−axis,φ:y−axis,κ:z−axis and the x-y-z convention for [**R**,T] building is incorporated. Formula (12) denotes the point-to-point observation equation used in optimization, where there are *C* pair-correspondences of source point to the target point.

(12) $$\min_{R,T}\sum_{i=1}^{C}{\left(\left(x_{i}^{t},y_{i}^{t},z_{i}^{t}\right)-\Psi_{R,T}\left(x_{i}^{s},y_{i}^{s},z_{i}^{s}\right)\right)}^{2}$$

Semantic point-to-point observation equation is defined by Equation (13), where there are $C^{l}$ correspondences of neighboring points with the same semantic label *l*.

(13) $$\min_{R,T}\sum_{i=1}^{C^{l}}{\left(\left(x_{i,l}^{t},y_{i,l}^{t},z_{i,l}^{t}\right)-\Psi_{R,T}\left(x_{i,l}^{s},y_{i,l}^{s},z_{i,l}^{s}\right)\right)}^{2}$$

Semantic labels are assigned during LiDAR data processing (Section 3.3).

#### 3.4.2. Semantic Point-to-Projection Observation Equation

Classification into planes and lines enables incorporating the point-to-projection observation equations. These observations are derived from matching points having the same semantic label. It means that observations are built from points with the same local shape characteristics. It is evident that once these projections are calculated using the above described point-to-point approach, they can be used as the observation equations. Look at the projection of point $P^{src,l}\left(x^{src,l},y^{src,l},z^{src,l}\right)$, which can be transformed to the global coordinate system as point $P^{src,g}\left(x^{src,g},y^{src,g},z^{src,g}\right)$ using matrix [**R**,T]. Thus,

(14) $$\left[\begin{array}{c} x^{src,g}\\ y^{src,g}\\ z^{src,g} \end{array}\right]=\Psi_{R,T}\left(x^{src,l},y^{src,l},z^{src,l}\right)=\left[R,T\right]\left[\begin{array}{c} x^{src,l}\\ y^{src,l}\\ z^{src,l} \end{array}\right]$$

to find point $P^{src,g}$ to line projection as $P^{proj,g}$ in the global reference system, line representation is used as target direction vector $V^{trg,ln}\left(x^{trg,ln},y^{trg,ln},z^{trg,ln}\right)$ and target point on line $P^{trg,g}\left(x^{trg,g},y^{trg,g},z^{trg,g}\right)$ is expressed in global reference system. Therefore, the point-to-line projection is as follows:

(15) $$P^{proj,g}=P^{trg,g}+\frac{a\cdot b}{b\cdot b}b,a=\left[\begin{array}{c} x^{src,g}-x^{trg,g}\\ y^{src,g}-y^{trg,g}\\ z^{src,g}-z^{trg,g} \end{array}\right],b=\left[\begin{array}{c} x^{trg,ln}\\ y^{trg,ln}\\ z^{trg,ln} \end{array}\right]$$

where (·) is a dot product.

To find point $P^{src,g}$ to plane projection as $P^{proj,g}$, the following plane equation is considered:

(16) $$ax+by+cz+d=0,∥\left[\begin{array}{ccc} a & b & c \end{array}\right]∥=1$$

$V^{pl}=\left(a,b,c\right)$ is the unit vector orthogonal to the plane, and *d* is the distance from the origin to the plane. It satisfies the following condition with the point in 3D space

(17) $$\left[\begin{array}{cccc} a & b & c & d \end{array}\right]\left[\begin{array}{c} x\\ y\\ z\\ 1 \end{array}\right]=0$$

Therefore, projection $P^{proj,g}$ can be computed with:

(18) $$P^{proj,g}=\left[\begin{array}{c} x^{src,g}\\ y^{src,g}\\ z^{src,g} \end{array}\right]-\left(\left[\begin{array}{c} x^{src,g}\\ y^{src,g}\\ z^{src,g} \end{array}\right]\cdot V^{pl}\right)V^{pl}$$

where (·) is a dot product. To build point-to-line projection or point-to-plane projection, observation Equation (13) can be incorporated.

#### 3.4.3. Relative Pose Observation Equation

Relative pose observation equation concerns a relative pose **P** (x,y,z,ω,φ,κ) from pose $A_{from}$ to pose $B_{to}$$\left(P=A_{from}^{-1}B_{to}\right)$ and a desired pose $P^{t}$; therefore optimization will converge by moving poses $A_{from}$ and $B_{to}$ to reach the desired relative pose $P^{t}$. To construct the observation equation, the function *m2v* is incorporated to compute vector (x,y,z,ω,φ,κ) from matrix **P** assuming Tait–Bryan angle convention. Therefore, the optimization problem is defined in Equation (19), where ($x_{i}^{t}$,$y_{i}^{t}$,$z_{i}^{t}$,$\omega_{i}^{t}$,$\varphi_{i}^{t}$,$\kappa_{i}^{t}$) is a target relative pose (the desired one) that the optimization is supposed to converge with.

(19) $$\min_{R^{A},T^{A},R^{B},T^{B}}\sum_{i=1}^{C}{\left(\left(x_{i}^{t},y_{i}^{t},z_{i}^{t},\omega_{i}^{t},\varphi_{i}^{t},\kappa_{i}^{t}\right)-m2v{\left(A_{from}^{-1}B_{to}\right)}_{i}\right)}^{2}$$

### 3.5. GNSS + INS Accuracy Assessment

Figure 2 shows the implementation of the proposed methodology for GNSS + INS accuracy assessment using LiDAR SLAM data alignment as a confirmation tool. Once mobile mapping data covering the expected region are collected, they are processed using methods described in Section 3.2 and Section 3.3. GNSS + INS data processing provides trajectories and accuracy assessment for each node of trajectory as one of the following classes (Class 1: 0–0.15 m, Class 2: 0.05–0.4 m, Class 3: 0.2–1.0 m, Class 4: 0.5–2.0 m, Class 5: 1.0–5.0 m, Class 6: 2.0–10.0 m). To confirm this accuracy assessment, LiDAR SLAM alignment is performed for all of these trajectories. This method provides an optimal solution guaranteeing no systematic drift by minimizing the distance between landmarks in aligned trajectories. Relative poses are calculated for all corresponding nodes between the input trajectories and the aligned ones. These relative poses are concatenated into histograms in Section 5.2; therefore, it is possible to quantitatively verify the percentage of the data set satisfying certain accuracy conditions defined as Classes 1–6. It was experimentally proven that the accuracy assessment provided by the NovAtel GNSS + INS processing tool is very similar to the SLAM output. This confirmed accuracy assessment can be used to consider the impact GNSS + INS positioning has on safety, as discussed in Section 6. The cause of SLAM errors is discussed as a real-world challenge in Section 4. Due to the volume of processed data and manual verification, SLAM errors are considered to have a minor impact on the overall confirmation of the accuracy assessment.

## 4. Real-World Challenges

**Figure 8:**
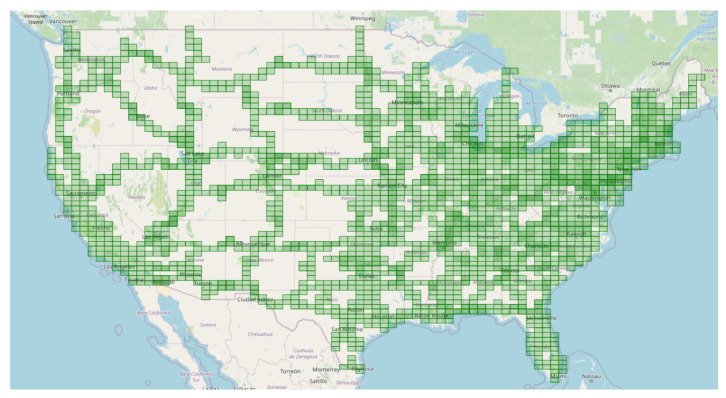
Real-world challenge: high volume of data covering the United States. Green rectangles correspond to visited regions by MoMa cars collecting data.

To reconstruct the map of a continent, e.g., North America, it is necessary to cope with many challenges caused by the volume of data (Figure 8) and errors related to raw data acquired at different times. The dominant issue is related to the gap between two time-intervals where data were acquired; thus, changes in the observed environment could occur, having a negative impact on SLAM convergence, and finally the result could yield a suboptimal solution.

**Figure 9:**
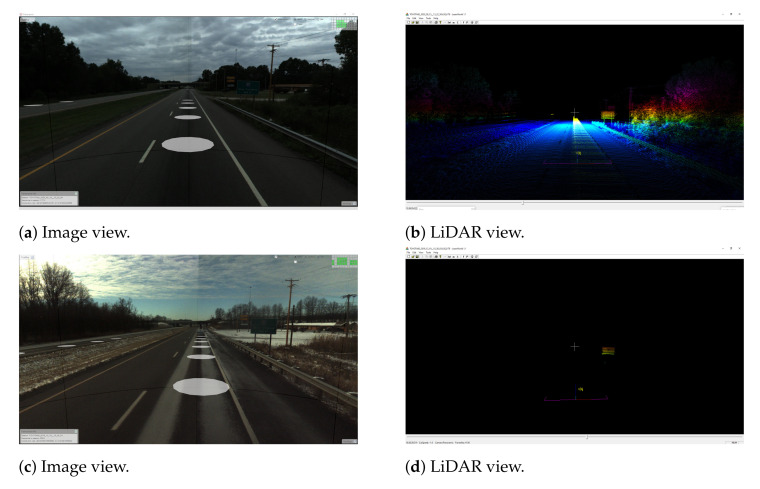
Real-world challenge: lack of LiDAR observations caused by environmental conditions; (**a**,**b**)—typical environmental conditions; (**c**,**d**)—winter conditions, only high reflective surfaces were detected by LiDAR.

**Figure 10:**
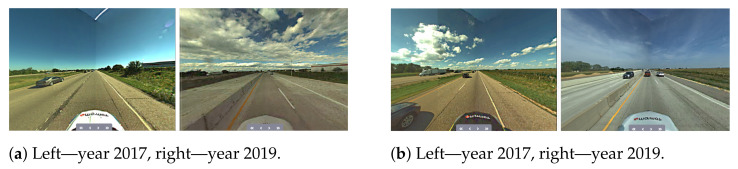
Real-world challenge—roadworks.

**Figure 11:**
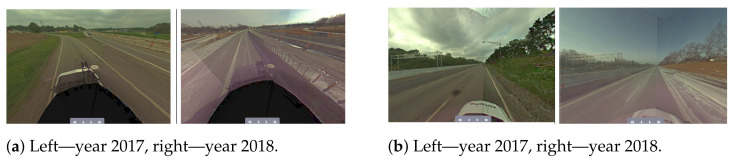
Real-world challenge—vegetation.

**Figure 12:**
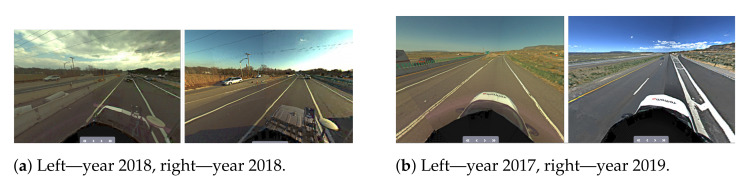
Real-world challenge—repainting.

**Figure 13:**
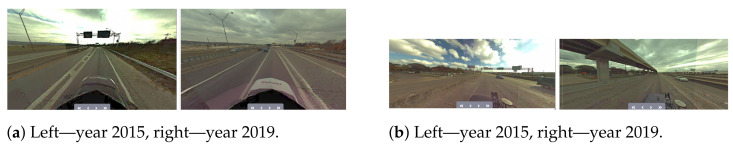
Real-world challenge—multi level changes.

Another challenge is related to having a sufficient coverage of the map; thus. it is evident that many places have to be observed (visited) many times to reduce the possible impact of factors such as noisy data, low-quality data and heavy traffic (Figure 5). The area is covered sufficiently when there are many overlaps from the LiDAR measurement point of view. As in many mobile mapping approaches, it is advised to guarantee at least 70% coverage (70% of LiDAR data from one trajectory can find correspondences to LiDAR data of other trajectories). The SLAM techniques require as good correspondences between observations as possible; thus, any disruptive information can affect the algorithm convergence, making it a suboptimal solution. After the experiment, it was found that in some cases, it was almost impossible to automatically find the correspondences between sessions where geometrical or other changes appeared. Therefore, the observed real-world challenges were classified into certain classes: (a) a lack of observations (Figure 9), (b) roadworks (Figure 10), (c) vegetation (Figure 11), (d) repainting (Figure 12) and (e) multi-level changes Figure 13. Such classification is proposed due to different impacts on the alignment process. In the current form of the SLAM implementation, these challenges are addressed by the motion model and GNSS + INS constraints that maintain the poses of the trajectories. The most challenging problem is the repainting of the lane dividers since a rather small discrepancy between the old and new paintings can affect alignment. Fortunately, this issue does not affect the entire accuracy assessment, since a large volume of data are processed and the probability of repainting all lane dividers in the whole United States region is rather low. Unknown obstacles are considered point-to-point observation equations.

## 5. Experimental Validation

### 5.1. Scope of Data Set

The scope of data covered by the experiment includes 32,785 trajectories collected in the USA by MoMa vans between 2016 and 2019. The total length of trajectories is 1,159,956.9 km, and 11,526,543 nodes were used in the analysis. Since calculations performed by SLAM take 200 times more 6DOF nodes, the result of optimizing 186.4 × 10${}^{9}$ parameters is reported. In Table 2, there is information collected about the distribution of the data source from the point of view of reported NovAtel accuracy. It can be observed that most of the accuracies of the input data are within the range of 0.0–1.0 m.

### 5.2. Results

The major issue within the context of large scale SLAM systems corresponds to the availability of the ground truth data. The methodology for evaluating such systems assuming the existence of the ground truth data source can be found in [61]. Since the only ground-truth information comes from input GNSS + INS data, it can be justified if SLAM move poses within a certain interval. In that sense, it is possible to justify how much SLAM had to move trajectories to reach the more consistent result. In Table 3, the results are summarized. For each category, the difference between GNSS + INS and SLAM results was computed as relative pose. These values were summarized in histograms; therefore, it is possible to justify the percentage of data maintaining the reported quality. An interesting observation is that results for the 2D error are more optimistic, therefore it is claimed that the post-processed GNSS + INS data are less precise in altitude. It can be observed in Table 3 that 52.5% of post-processed GNSS + INS data of class 1 are moved not more than 15 cm by SLAM according to the 3D error, 81.7% of data of class 2 are moved not more than 0.4 m by SLAM according to the 3D error, and 91.7% of data of class 3 are moved not more than 1.0 m by SLAM according to the 3D error. As shown by the 2D error, 84% of data of class 1 are moved by no more than 15 cm by SLAM. Therefore, it can be seen that the accuracy of altitude is much worse than the accuracy of longitude and latitude. This observation must be taken into consideration during navigation on multi-level roads. The most problematic angle is roll, since it corresponds mainly with long straight trajectories where this angle is difficult to measure by IMU; therefore, SLAM produces the most significant corrections. An interesting observation is that there are many situations where the accuracy of post-processed GNSS + INS data is better than reported by NovAtel. Manual inspection was performed using HD map of the SLAM alignment and based on the inspection it is concluded that this technique can confirm the accuracy assessed by NovAtel algorithm, and it can improve trajectories even when some minor errors of SLAM appear. The causes of these errors were collected as challenges in Section 4. The investigation of SLAM errors will be the focus of our future research.

**Figure 14:**
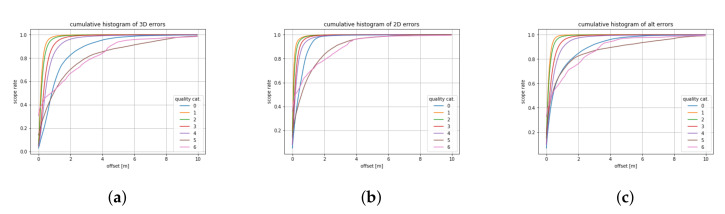
Histograms of 3D (**a**), 2D (**b**) and altitude (**c**) errors measured as cumulated relative poses between GNSS + INS and SLAM alignment.

Figure 14 demonstrates the quantitative results collected in Table 3.

## 6. Impact of GNSS + INS Positioning on Safety

For the impact of GNSS positioning on safety, the following aspects are considered: a hypothetical Mid-Size vehicle type, a mean distance between lanes as 3.6 m (limited-access highways in the United States of America) and Lateral Alert Limit as 0.72 m and Longitudinal Alert Limit as 1.40 m according to [1] (as a reference, reported values of accuracy and alert limits of relative positioning for different types of the vehicle versus map are shown in Table 1). This scenario is the most optimistic one since a small vehicle is considered. It is assumed that, during an autonomous drive, there is the same GNSS + INS system for positioning and that the real-time calculations have the same accuracy as in postprocessing presented in the experiment. From all trajectories (total length: 1,159,956 km), 710,958 km is class 1 (61.3%), 378,453 is class 2 (32.63%) and 49,335 is class 3 (4.25%). The defined accuracy by NovAtel for class 1 is (0.0–0.15 m) for class 2 (0.05–0.4) and class 3 (0.2–1.0); thus, if the entire data set can reach such classes, it can be considered as having a high probability of satisfying the Alert Limits for Mid-Size (width 1.85 m, length 4.87 m) vehicle localization moving on limited-access highways in the United States of America. In our case, it is calculated as 14,132 km of class 4 (1.22%) 715 km of class 5 (0.006%) and 59 km of class 6 (0.005%). To summarize, 98.17% of processed data belong to classes 1–3, while 1.83% of data do not belong to classes 1–3 and could cause exceeding the alert limits. To verify these classes, further calculations are performed related to the alignment of the trajectories as part of the proposed methodology. Almost 99% of data satisfy NovAtel 1–3 classes; therefore, this additional calculation confirms the fact of more than 1% of data could cause hitting alert limits for a Mid-Size vehicle. For the larger vehicles, e.g., for six-Wheel Pickup (width 2.03–2.43 m, length 5.32–6.76 m), the Lateral Alert Limit is 0.4 m; thus, according to the proposed methodology only around 95% of data satisfy it.

## 7. Conclusions

This paper concerns a new methodology for accuracy assessment of a global positioning system at continent scale for assessing autonomous driving safety. Safety is addressed as an alert limit for the defined geometry of the problem, where the aim is to maintain knowledge that the vehicle (its bounding box) is within its lane. Hypothetical Mid-Size and 6-Wheel Pickup types of vehicles were considered, and the mean distance between lanes as 3.6 m as representative boundaries of the vehicles moving on the limited-access highways in the United States of America. A new methodology of the global positioning accuracy assessment is proposed, incorporating mapping systems performing road surveys covering United States region in 2016–2019 period. It is composed of six elements: (1) mobile mapping system with minimal setup, (2) global positioning data processing, (3) LiDAR data processing, (4) alignment algorithm, (5) accuracy assessment confirmation and (6) autonomous driving safety analysis. It relates to the main goal of measuring the impact of global positioning on autonomous driving safety, assessed as calculation of GNSS + INS accuracy confirmed with additional trajectory alignment. The novelty of the approach is the large-scale evaluation based on massive mobile mapping data, GNSS + INS processing for accuracy assessment and introducing LiDAR SLAM-based data alignment to confirm accuracy. The research challenge was to assess the positioning accuracy of the moving cars assuming full coverage of limited-access highways in the United States of America. The expected coverage limits the possibility of repetitive measurements and introduces an important challenge of the lack of availability of the ground truth data. Therefore, the state-of-the-art methodology is not applicable for this particular application, and a novel approach is proposed. The idea is to align all trajectories using LiDAR to confirm the accuracy reported by the state-of-the-art GNSS + INS data processing performed at a large scale. For this reason, it is investigated how to use LiDAR metric measurements for data alignment implemented using SLAM (Simultaneous Localization and Mapping) assuring no systematic drift thanks to applying GNSS + INS constraints. The SLAM implementation uses state-of-the-art observation equations and the Weighted Nonlinear Least Square optimization technique capable of integration of required constraints. The methodology was verified experimentally using arbitrarily chosen measurement instruments (NovAtel GNSS + INS, LiDAR Velodyne HDL32) mounted onto mobile mapping systems. The proposed methodology extends the existing methods of global positioning system accuracy assessment with the focus on realistic conditions and full area coverage. The impact of the global positioning system accuracy on autonomous car safety is discussed. It is shown that 99% of the assessed data satisfied safety requirements (driving within lanes of 3.6 m) for Mid-Size vehicles and 95% for 6-Wheel Pickup. The conclusion is that this methodology has great potential for global positioning accuracy assessment at the global scale for autonomous driving applications. Further research is required to solve challenges affecting data alignment as the reference tool for accuracy confirmation.

## Figures and Tables

**Table 1:** Localization requirements for US freeway operation with interchanges. This assumes minimum lane widths of 3.6 m and allowable speeds up to 137 km/h (85 mph).

Vehicle Type	Lateral Alert Limit [m]	Longitudinal Alert Limit [m]
Mid-Size	0.72	1.40
6-Wheel Pickup	0.40	1.40

**Table 2:** Quality categories distribution in analyzed data.

NovAtel Quality	Distance [km]	% of Total	3D Accuracy (m)
1	710,958	61.29	0.0–0.15
2	378,453	32.63	0.05–0.4
3	49,335	4.25	0.2–1.0
4	14,132	1.22	0.5–2.0
5	715	0.06	1.0–5.0
6	59	0.01	2.0–10.0

**Table 3:** Qualities verified using SLAM.

Quality	3D Accuracy (m)	% 3D Diff	% 2D Diff	% Altitude Diff
1	0.0–0.15	52.5	84.0	65.6
2	0.05–0.4	81.7	93.7	88.3
3	0.2–1.0	91.7	97.8	94.5
4	0.5–2.0	96.1	99.0	97.3
5	1.0–5.0	88.6	97.8	91.6
6	2.0–10.0	98.4	99.5	99.2

## Data Availability

Not applicable.

## Abbreviations

The following abbreviations are used in this manuscript:

LiDAR	Light Detection and Ranging
GPS	Global Positioning System
GNSS + INS	Global Navigation Satellite System + Inertial Navigation System
SLAM	Simultaneous Localization and Mapping
RANSAC	Random Sample Consensus
